# L-Carnitine Improves Muscle Nutrient Metabolism and Intestinal Health in High-Fat-Fed Carp (*Cyprinus carpio*)

**DOI:** 10.1155/anu/5623889

**Published:** 2025-01-09

**Authors:** Xianglin Cao, Rongjie Yuan, Yi Guo, Mengtao Jia, Yinyin Wei, Jiameng zhou, Han Cui, Baohua Li, Jianjun Chen

**Affiliations:** ^1^College of Fisheries, Henan Normal University, Xinxiang 453007, China; ^2^College of Life Science, Henan Normal University, Xinxiang 453007, China

**Keywords:** intestinal health, intestinal metabolites, intestinal microbiology, muscle quality, nutrient metabolism

## Abstract

L-Carnitine is widely recognized for its involvement in lipid metabolism, but its effects on muscle quality and gut health in carp have not been well studied. The research aimed to investigate how L-carnitine influences muscle quality and intestinal health in high-fat-fed carp. The study was separated into four groups that received either the standard diet, a high-fat diet (HFD), or a HFD supplemented with 500 mg/kg L-carnitine (LLC), or a HFD supplemented with 1000 mg/kg L-carnitine (HLC) for 56 days. L-Carnitine was found to significantly reduce blood lipid levels. In addition, L-carnitine increased the crude protein content and decreased the crude fat content of high-fat-fed carp muscle while improving muscle fiber morphology and muscle quality. L-Carnitine increased the expression of genes related to intestinal tight junction proteins (*claudin-2*, *occludin*, and *zo-1*), improved the expression of genes related to intestinal inflammation, and enhanced the physical barrier function and organization of the intestine. Analysis of intestinal flora and intestinal metabolites showed that L-carnitine increased the diversity of the intestinal flora, increased the abundance of *Cetobacterium*, and influenced intestinal levels of bile acids, arachidonic acid, and tryptophan-related metabolites. In conclusion, supplementation with 1000 mg/kg L-carnitine improved muscle quality and intestinal health significantly in high-fat-fed carp by regulating muscle nutrient metabolism and intestinal flora.

## 1. Introduction

Numerous studies are being conducted to produce the highest quality products for human consumption at the lowest possible cost in today's rapidly growing aquaculture industry [1]. It is crucial to ensure the healthy state of fish and the quality of fish meat while reducing costs. Strategies that have been proposed to solve this problem include finding some alternative feed ingredients and adding some plant extracts, probiotics, feed additives, etc. to the feed [2].

Proteins and lipids are important nutritional sources in fish diets; however, proteins are much more expensive than lipids [3]. To economize on the cost of feeds, some studies have proposed strategies to replace some of the proteins in feeds with lipid-based ingredients [4]. High-fat diets (HFDs) are widely employed for their ability to preserve dietary protein and improve feed efficiency [5]. However, it can disrupt lipid metabolism in zebrafish (*Danio rerio*) and inhibit muscle protein synthesis in Nile tilapia (*Oreochromis niloticus*) [6, 7]. Additionally, by damaging the structure and normal microbiome of the intestine, HFDs could damage gut health [8, 9]. Therefore, looking for additives that can mitigate the negative effects of high-fat feeding is important.

L-Carnitine is critical to lipid metabolism by promoting the conversion of more lipids into energy [10]. L-Carnitine has multiple important applications in aquaculture. For example, it serves as a synthetic substrate for carnitine palmitoyltransferase I (CPT-I) and carnitine-acylcarnitine transporter (CACT), regulates lipid metabolism, and promotes β-oxidation for energy production [11]. Besides reducing body fat, L-carnitine also reduces the level of inflammation in the body [12]. L-Carnitine improved the antioxidant capacity and reduced the inflammation of black seabream [13]. Furthermore, L-carnitine increases fatty acid oxidation in skeletal muscle, maximizes lipid utilization in animals, and increases muscle protein levels [14]. Feeding 500 mg/kg L-carnitine significantly improved muscle texture in largemouth bass [15]. Numerous research studies have demonstrated L-carnitine can alleviate lipid accumulation from high-fat feeding by promoting lipolysis, but no study has yet confirmed whether L-carnitine improves muscle quality and intestinal health in high-fat-fed carp.

Carp (*Cyprinus carpio*) represents a significant fish species that is economically farmed in China. In addition, carp have been the subject of much study and have a large amount of background information and research base. The impact of L-carnitine on muscle quality in high-fat-fed carp was evaluated by observing muscle fiber morphology, measuring lipid metabolism and protein metabolism-related gene expression in muscle, detecting fatty acid composition in muscle, and determining muscle texture. The ameliorative impact of L-carnitine upon the intestinal physical barrier of high-fat-fed carp was evaluated by observing the intestinal morphology and determining the gene expression that is related to inflammation, apoptosis, autophagy, antioxidant, and tight junction proteins. Subsequently, to further investigate how L-carnitine affects intestinal health, changes in intestinal microbiota and metabolites were analyzed. Finally, the intestinal flora and intestinal metabolites were analyzed in association with other indicators tested to investigate the possible mechanisms by which L-carnitine improves high-fat carp intestinal health and muscle quality.

## 2. Materials and Methods

### 2.1. Experimental Design

The carps employed for this experiment were obtained from a fishery in Xinxiang, China. All fish were placed in tanks for 4 weeks to acclimatize before starting the formal experiment. The commercial diet was fed during a 4-week acclimation period. After 4 weeks of domestication, 360 carps (16.44 ± 2.13 g) were separated into four groups: (1) control, (2) HFD, (3) HFD supplemented with 500 mg/kg L-carnitine (LLC), and (4) HFD supplemented with 1000 mg/kg L-carnitine (HLC), according to their initial mean body weight. The feeding experiment lasted for 8 weeks. The purity of L-carnitine used in this experiment was 98%, purchased by Aladdin.  Table 1 describes the specific formulation of the diet. The feed manufacturing process involves first determining the feed formula, followed by crushing the ingredients to ensure they are evenly distributed during the subsequent mixing process. The weighed ingredients were then placed in a mixer and thoroughly mixed to ensure that the various ingredients were evenly distributed throughout the feed. Once mixing was complete, the mixed feed was processed into 3-mm pellets using a pelletizer. The feeds were dried and kept at −20°C for later use. One group consisted of three 300-L tanks, each containing 30 carps. The experimental carps were reared using a circulating water system with daily feeding at 10:00, 14:00, and 18:00 until the carps were visibly satiated. Throughout the feeding period, the water temperature remained between 23 and 25°C, the dissolved oxygen concentration was constantly held above 5.0 mg/L, the ammonia nitrogen concentration was controlled below 0.5 mg/L, and a natural photoperiod (12 h light/dark cycle) was maintained.

When the culture experiment was completed, the carp were starved for 12 h and then drugged with MS-222. Then, according to previous studies, the weight, hepatopancreas weight, and viscera weight of the fish were determined, and specific growth indicators were calculated [16]. Tail venous blood was collected for serum biochemical indices; muscle was collected for section preparation, fatty acid composition assay, RNA extraction, and texture determination; intestinal tissues were separated for section preparation and RNA extraction; and intestinal contents were collected for intestinal microbial and metabolite analysis.

### 2.2. Determination of Feed Composition

The nutrient composition of diets and muscle were analyzed by standard laboratory procedures [17]. These included the analysis of crude protein by the Kjeldahl method and the determination of crude lipid content using Soxhlet extraction. The moisture content was calculated through desiccation to stable weight at 105°C.

### 2.3. Measurement of Growth Performance

At the end of the culture experiment, the fish were fasted for 12 h. The carp were then anesthetized with MS-222. Subsequently, the weights of the carp's viscera, hepatic, and whole body were measured using an electronic balance. Growth performance-related indices were calculated according to the method described in our previous study [16].

### 2.4. Histomorphological Analysis

The muscles and intestines were collected after the fish were anesthetized and then washed in PBS. The tissues were then immobilized in paraformaldehyde for 12 h, dehydrated in alcohol, rendered clear in xylene, encapsulated in paraformaldehyde, and sectioned by the microtome (Thermo Scientific Microm HM 340E). After completing this procedure of sectioning, muscle sections and intestinal sections were stained with H&E dye, and then all sections were visualized with a microscope after sectioning was completed (Nikon Eclipse E400; Tokyo, Japan). According to the previous method, the muscle fiber diameter within the 0.30 mm^2^ area was calculated using ImageJ. The area of muscle fibers was first determined, and then the diameter of muscle fibers was calculated [18]. Indicators related to intestinal tissues were quantified using ImageJ.

### 2.5. Serum Biochemical Analysis

The collected blood was centrifuged at 590×*g* for 10 min at low temperature to collect serum. The levels of total triglycerides (TGs, BC0625), total cholesterol (T-CHO, BC1985), low-density lipoprotein cholesterol (LDL-C, BC5335), high-density lipoprotein cholesterol (HDL-C, BC5325), malondialdehyde (MDA, BC0025), catalase (CAT, BC0205), glutathione (GSH, BC1175), alanine aminotransferase (ALT, BC1555), aspartate aminotransferase (AST, BC1565), acid phosphatase (ACP, BC2135), and alkaline phosphatase (AKP, BC2145) were determined using kits from Solarbio (Beijing, China).

### 2.6. Muscle Fatty Acid Composition Analysis

The carp muscles were first freeze-dried. Then 0.1 g of the dried sample was taken and prepared as before [19]. The samples were mixed with 5-mL chloroform–methanol mixture, and then the tubes were fixed on a shaker to shake well for 2 h. The residue in the homogenate was removed using quantitative filter paper, while the filtered liquid sample was mixed with 4 mL of distilled water and centrifuged the samples on a rotating speed at 860×*g* for 5 min. A stratification of the solution can be observed after centrifugation. Take 1 mL of the lower solution and evacuate it under negative pressure in a water bath at 40°C. Next, 1 mL of hexane and potassium hydroxide–methanol solution were added to the negative pressure drained sample, which was left for 30 min and then shaken for 2 h. Wherewith, the tube was added with 2 mL deionized water, left to stratify, and was centrifuged at 860×*g* for 10 min. Finally, all samples were filtered into injection vials using 0.22-μm filter membranes. The prepared samples were tested by gas chromatography–mass spectrometry (Agilent Technology Corporation 7890-5977 A, USA).

### 2.7. Detection of Muscle Texture

The fresh muscle from the back of the carp was taken and cut into 1 cm × 1 cm × 0.5 cm blocks for measurement, detecting muscle cohesiveness, chewiness, adhesiveness, gumminess, springness, and hardness with texture analyzer (TA-XT 2i). The parameters were obtained from cylindrical probes with a diameter of 8 mm, a force sensing element with a range of 25 N, a detection speed of 30 mm/min, deformation of 60%, a starting force of 0.1 N, and interval time of 2 s.

### 2.8. Quantitative Real-Time PCR

The muscles and intestinal tissues were collected and kept at −80°C. Add 0.1 g sample to an enzyme-free test tube, and insert 1 mL of TRIzol reagent to extract total RNA. The quality and concentration of RNA were assessed using the microspectrophotometer. The PrimerScript RT Kit was used to synthesize the cDNA. The primers involved in the experiments are listed in Table S1. The procedure is as follows: predenaturation at 95°C for 10 min, denaturation at 95°C for 10 s, and annealing at 60°C for 30 s, and this cycle was repeated 40 times. Next, a dissolution profile was performed: 95°C for 15 s, 65°C for 60 s, and again at 95°C for 15 s. Finally, a cooling step was performed and held at 37°C for 30 s. The 40 s was used as a reference gene because of its stable nature. The experiments were analyzed using the Light 96 real-time PCR detection system, and the results were calculated using the 2^−*ΔΔ*CT^ method [20].

### 2.9. Sequencing of Intestinal Flora

After the fish were anesthetized, the mid and hindgut contents were taken in three replicates per experimental group. The DNA extraction kit from TIANGEN Biotechnology Ltd. in Beijing, China, was utilized to extract microbial genomic DNA from intestinal contents. Assess the concentration of DNA using a microspectrophotometer. Finally, the gel was recovered for DNA purification and subjected to sequence analysis. The V3–V4 region of the bacterial 16S rRNA gene was amplified using the specific primer 338F/806R. Subsequently, the samples were subjected to sequence measurement and gene library construction using the MajorBio cloud platform. Additional examination of the sequencing data was conducted utilizing the MajorBio platform (www.majorbio.com).

### 2.10. Intestinal Metabolite Analysis

After weighing an appropriate amount of sample, 400 μL of methanol solution was added. Samples were crushed using a high-throughput tissue crusher, vortexed, mixed, and sonicated on ice. After leaving the sample at −20°C for 30 min, the sample was centrifuged at 13680×*g* for 15 min. The supernatant was collected, filtered, and transferred to the injection bottle to wait for LC-MS analysis. A BEH C18 column was used in this experiment, with 0.1% formic acid aqueous solution in A phase and acetonitrile/isopropanol solution (1/1) in B phase. The samples were separated using the gradient elution method, and the time taken for each sample was 16 min. During instrumental analysis, one QC sample was added for every eight samples.

### 2.11. Data Analysis

All data are presented as mean ± SEM. Tukey test was employed for multiple group comparisons. GraphPad Prism (version 7.0) was utilized to perform the one-way ANOVA in this trial. Multiple group comparisons were made by Tukey's test. In this study, a difference was defined as significant at *p* < 0.05. Different letter marks represent significant differences. For microbiome analysis, β diversity was explored with weighted UniFrac and visualized by using PLS-DA. Use the Kruskal–Wallis *H* test to detect differences in intestinal microbiota between groups. Metabolites were statistically analyzed with R software. The data were annotated by comparison with the KEGG database.

## 3. Results

### 3.1. Effect of L-Carnitine on the Growth Performance of High-Fat-Fed Carp

Table 2 shows the growth indicators of carp in each group. High-fat feeding significantly increased the final body weight of carp. However, L-carnitine did not significantly affect body weight in high-fat-fed carp. Interestingly, supplementation with 1000 mg/kg L-carnitine decreased the hepatopancreatic body index in carp significantly (*p* < 0.05).

### 3.2. L-Carnitine Meliorated Serum Biochemical Parameters in High-Fat-Fed Carp

**Figure 1:**
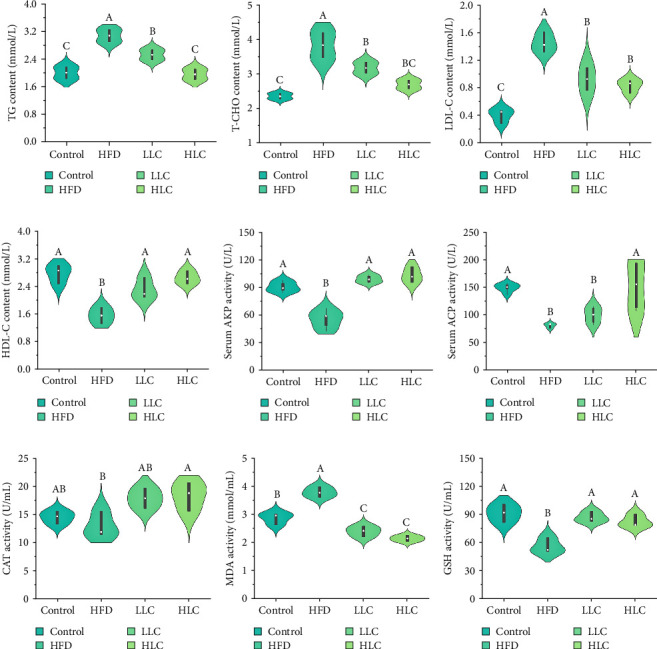
L-Carnitine improved serum biochemical indices, immunoenzymes, and antioxidant enzymes in high-fat common carp. (a–d) The level of blood lipid. (e–g) The activity of AKP, ACP, and CAT. (h, i) The content of MDA and GSH. Different letters indicate significant differences (*p* < 0.05). ACP, acid phosphatase; AKP, alkaline phosphatase; CAT, catalase; GSH, glutathione; HDL-C, high-density lipoprotein cholesterol; HFD, high-fat diet; HLC, HFD supplemented with 1000 mg/kg L-carnitine; LDL-C, low-density lipoprotein cholesterol; LLC, HFD supplemented with 500 mg/kg L-carnitine; MDA, malondialdehyde; T-CHO, total cholesterol; TG, triglyceride.

Compared with high-fat-fed carp, L-carnitine reduced TG, T-CHO, and LDL-C levels and significantly increased HDL-C levels (*p* < 0.05) (Figure 1). In addition, L-carnitine reduced the activity of serum ALT and AST in high-fat-fed carp (Figure S1). Furthermore, L-carnitine improved the antioxidant and immune properties of carp by significantly reducing serum MDA levels and increasing ACP, AKP, CAT, and GSH activities in high-fat-fed carp.

### 3.3. L-Carnitine Ameliorated Muscle Tissue Morphology and Muscle Composition in High-Fat-Fed Carp

**Figure 2:**
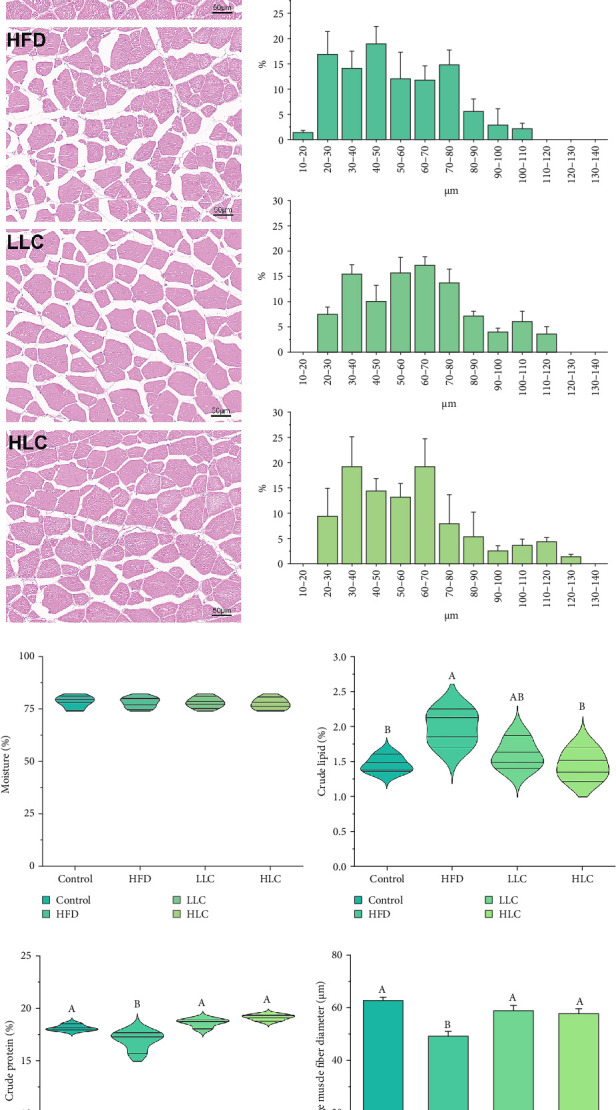
L-Carnitine improves muscle tissue morphology and muscle composition in high-fat carp. (a) Muscle tissue morphology. (b) Myofiber diameter distribution. (c) Moisture. (d) Crude lipid content. (e) Crude protein content. (f) Average myofiber diameter. Scale bar = 50 μm. Different letters indicate significant differences (*p* < 0.05). HFD, high-fat diet; HLC, HFD supplemented with 1000 mg/kg L-carnitine; LLC, HFD supplemented with 500 mg/kg L-carnitine.

To examine the effects of L-carnitine on muscle, muscle histomorphometry and nutrient composition were evaluated separately. The diameter of myofibers decreased, and the spacing of myofibers increased in carp after 8 weeks of feeding HFDs (Figure 2a,b,f). Furthermore, compared to high-fat-fed carp, L-carnitine supplementation significantly increased muscle fiber diameter in carp (Figure 2f). The addition of 1000 mg/kg L-carnitine significantly increased the crude protein content of the muscle, while it decreased the crude lipid content of the muscle (*p* < 0.05) (Figure 2c–e). This indicates that L-carnitine will improve muscle tissue morphology and composition in high-fat-fed carp.

### 3.4. L-Carnitine Improved the Expression of Genes Related to Muscle Nutrient Metabolism in High-Fat-Fed Carp

**Figure 3:**
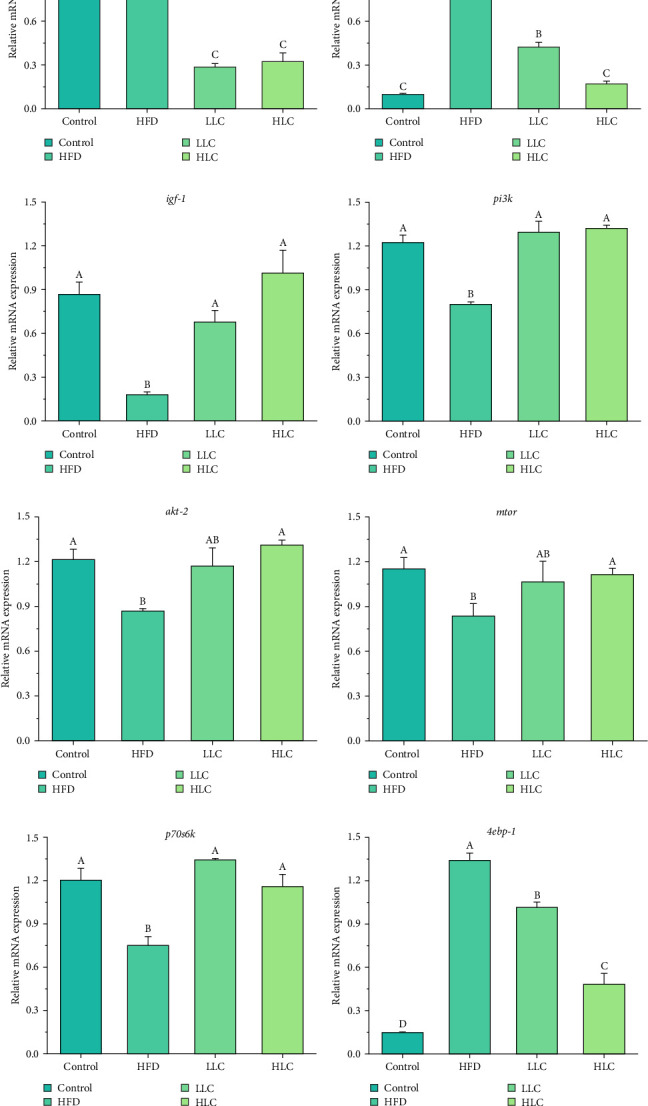
L-Carnitine regulated the expression of genes related to muscle nutrient metabolism in high-fat-fed carp. (a–f) Lipid metabolism-related genes. (g–n) Protein metabolism-related genes. (o–p) Amino acid metabolism-related genes. Different letters indicate significant differences (*p* < 0.05). HFD, high-fat diet; HLC, HFD supplemented with 1000 mg/kg L-carnitine; LLC, HFD supplemented with 500 mg/kg L-carnitine.

To investigate the reasons for the altered muscle composition by L-carnitine, we analyzed the nutrient metabolism of muscle tissue in each group (Figure 3). In comparison with the high-fat feeding group, L-carnitine significantly reduced the levels of protein degradation-related genes (*4ebp-1*, *foxo3a*, and *murf-1*), lipid synthesis-related genes, and amino acid catabolism-related genes (*gdh* and *bckdh*), while the levels of protein synthesis-related genes and lipolysis-related genes (*pparα*, *lpl* and *cpt-1a*) were increased (*p* < 0.05) in the muscles of carp. The results indicated that L-carnitine could improve muscle nutrient metabolism in high-fat-fed carp.

### 3.5. L-Carnitine Modified Fatty Acid Composition of Muscle in High-Fat-Fed Carp

Table 3 shows the influence of L-carnitine on the fatty acid composition of muscle from high-fat-fed carp. High-fat feeding significantly increased the n-3 PUFA content and decreased the n-6 PUFA content in muscle, whereas L-carnitine significantly increased the n-3 PUFA content and decreased the n-6 PUFA content in the muscle of high-fat-fed carp (*p* < 0.05). This suggests that L-carnitine changed the fatty acid composition of high-fat-fed carp muscle.

### 3.6. L-Carnitine Enhanced Muscle Structure in High-Fat-Fed Carp

**Figure 4:**
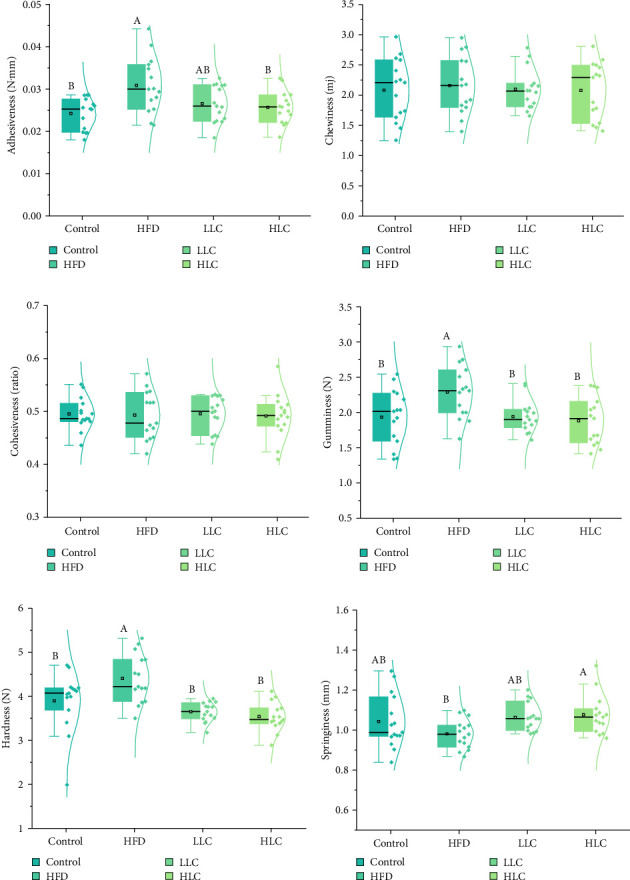
L-Carnitine improves muscle texture in high-fat-fed carp. (a) Adhesiveness. (b) Chewiness. (c) Cohesiveness. (d) Gumminess. (e) Hardness. (f) Springiness. Different letters indicate significant differences (*p* < 0.05). HFD, high-fat diet; HLC, HFD supplemented with 1000 mg/kg L-carnitine; LLC, HFD supplemented with 500 mg/kg L-carnitine.

Since changes in muscle composition affect muscle texture, we examined muscle texture in carp (Figure 4). It can be seen that high-fat feeding increased the adhesiveness, gumminess, and hardness of carp muscles, while it did not affect the springiness, chewiness, and cohesiveness. Compared to the high-fat feeding group, the addition of 500 mg/kg L-carnitine significantly decreased the adhesiveness and hardness but did not significantly affect the adhesiveness, chewiness, cohesiveness, and springiness (*p* < 0.05) of the carp muscles. Compared with the high-fat feeding group, the addition of 1000 mg/kg L-carnitine significantly (*p* < 0.05) decreased the adhesiveness, gumminess, and hardness and increased the springiness of carp muscles but did not affect the chewiness and cohesiveness.

### 3.7. L-Carnitine Alleviated Intestinal Damage and Boosted Intestinal Health in High-Fat-Fed Carp

**Figure 5:**
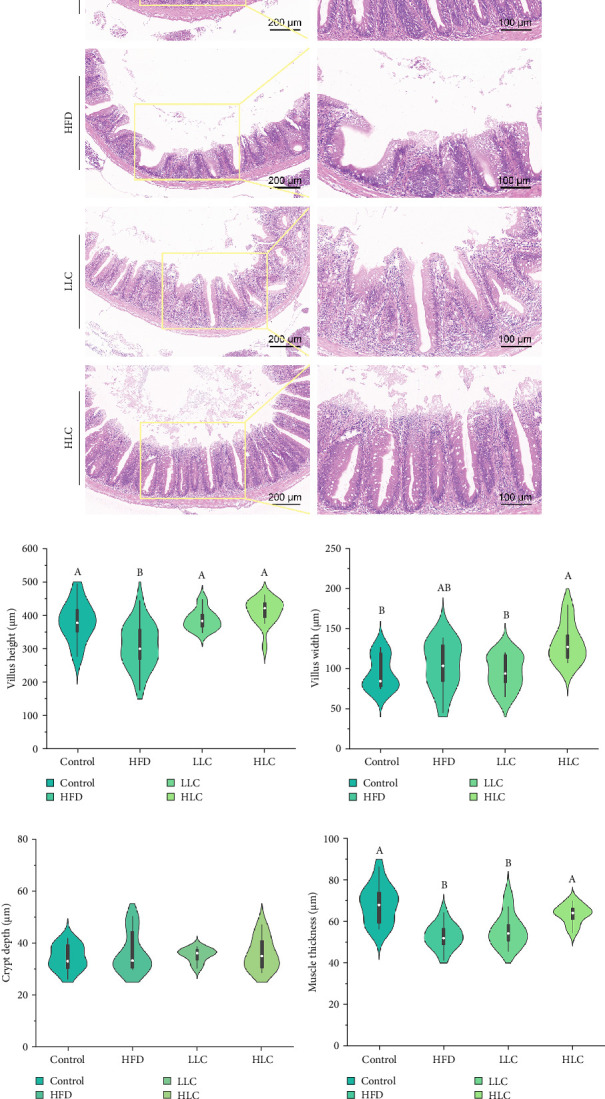
L-Carnitine improved intestinal histomorphology in high-fat-fed carp. (a) H&E-stained sections of the intestine; (b)villus height; (c) villus width; (d) crypt depth; and (e) muscle thickness. Different letters indicate significant differences (*p* < 0.05). HFD, high-fat diet; HLC, HFD supplemented with 1000 mg/kg L-carnitine; LLC, HFD supplemented with 500 mg/kg L-carnitine.

To investigate the impact of L-carnitine on intestinal tissue morphology in high-fat-fed carp, we prepared H&E-stained sections of the intestine. It can be seen that high-fat feeding significantly altered the histomorphology of the intestine, as evidenced by the reduction in the muscle layer thickness and the significant reduction in the height of the intestinal villi (*p* < 0.05; Figure 5a,b,e). In addition, we found that the addition of 500 mg/kg L-carnitine increased intestinal villi height in fat-fed carp (*p* < 0.05; Figure 5b). The addition of 1000 mg/kg L-carnitine significantly increased muscle layer thickness, height, and width of intestinal villi in high-fat-fed carp (*p* < 0.05; Figure 5b–e).

**Figure 6:**
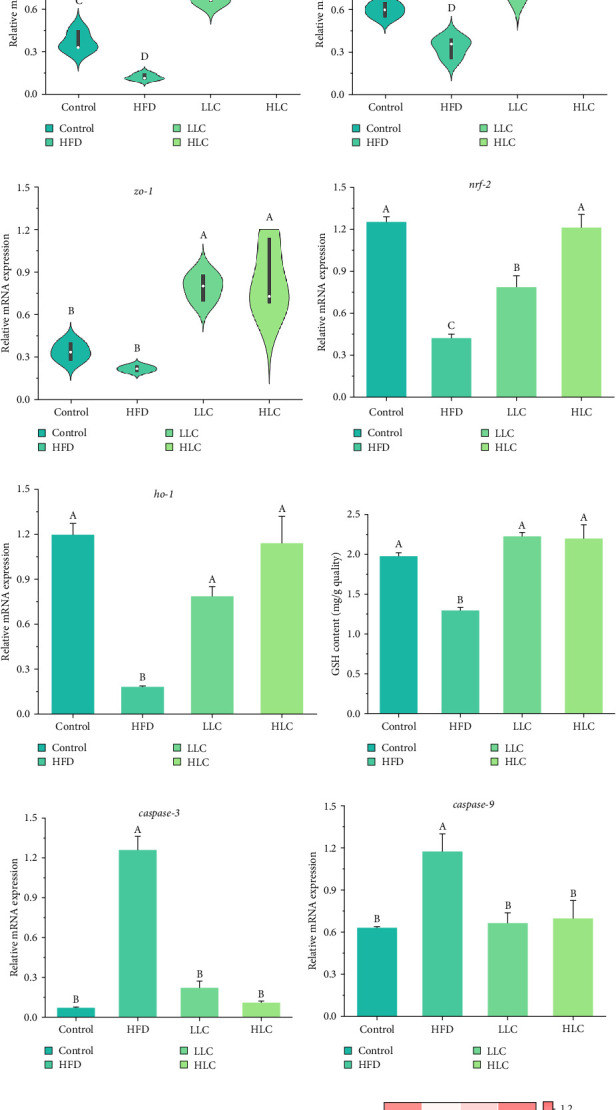
L-Carnitine increased mucus and tight junctions, promoted autophagy, improved antioxidant properties, and reduced apoptosis and inflammation in the gut of high-fat-fed carp. (a) AB-PAS-stained sections of the intestine. (b) Percentage of mucus. (c–e) Tight junction-related genes. (f, g) Antioxidant-related genes. (h) GSH content. (i–k) Apoptosis-related genes. (l) The expression levels of intestinal autophagy-related and inflammation-related genes. Different letters indicate significant differences (*p* < 0.05). GSH, glutathione; HFD, high-fat diet; HLC, HFD supplemented with 1000 mg/kg L-carnitine; LLC, HFD supplemented with 500 mg/kg L-carnitine.

The influence of L-carnitine on the intestine barrier, antioxidants, autophagy, apoptosis, and inflammation in high-fat-fed carps was then explored (Figure 6). Firstly, high-fat feeding induces a reduction in intestinal mucus, which was significantly ameliorated by L-carnitine (Figure 6a,b). Furthermore, L-carnitine significantly increased the expression of *claudin-2*, *occludin*, and *zo-1* (*p* < 0.05) (Figure 6c–e). Additionally, L-carnitine significantly reduced the expression of apoptosis-, inflammation-, and autophagy-related genes (*p62*) and increased the expression of antioxidant-related genes, autophagy-related genes (*atg-4 b*, *atg-12*, and *lc3ii*), inflammation inhibitory factor (*il-10*), and apoptosis inhibitory factor (*bcl-2*) and GSH content (*p* < 0.05) (Figure 6f–l).

### 3.8. L-Carnitine Restored Intestinal Microbial Dysfunction Induced by High-Fat Feeding in Carp

**Figure 7:**
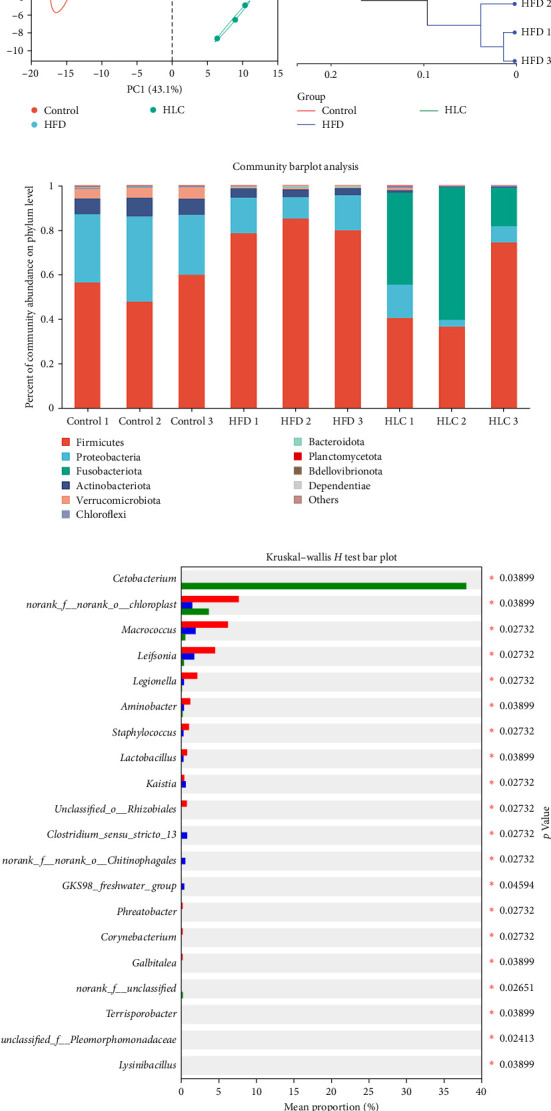
L-Carnitine improved gut microbiota in high-fat-fed carp. (a) Venn diagram. (b) Shannon index. (c) Chao1 index. (d) Distance box plot. (e) PCA on the OTU level. (f) Hierarchical clustering tree on OTU level. (g, h) Flora composition at the phylum and genus level. (i) PICRUSt analysis. Different letters indicate significant differences (*p* < 0.05). *⁣*^*∗*^*p* < 0.05. HFD, high-fat diets; HLC, HFD supplemented with 1000 mg/kg L-carnitine; PCA, principal component analysis.

From these results, it was found that the high-fat-fed group supplemented with 1000 mg/kg L-carnitine was more effective in restoring muscle quality and gut morphology in high-fat-fed carp compared with the high-fat-fed group supplemented with 500 mg/kg L-carnitine. Therefore, to investigate whether L-carnitine alleviation of intestinal health and regulation of muscle quality were related to intestinal flora and intestinal metabolites, we analyzed the intestinal flora and intestinal metabolite composition of carp in the 1000 mg/kg L-carnitine-added group. The results of Venn diagrams and distance box plots illustrated that the intestinal flora compositions of the three groups were significantly different, and the differences between the groups were significantly greater than those within the groups (Figure 7a,d). Additionally, the α-diversity of gut microflora was assessed by calculating the Chao1 index and Shannon index, and the prolonged high-fat feeding significantly (*p* < 0.05) decreased the Shannon index of carp gut microbiota. However, L-carnitine significantly increased the Shannon index (Figure 7b,c). The results of principal component analysis (PCA) and hierarchical clustering tree showed that both high-fat feeding and L-carnitine altered the structure of the intestinal microbiota (Figure 7e–f). At the phylum level, it showed that high-fat feeding increased the abundance of Firmicutes, whereas the addition of L-carnitine decreased the abundance of Firmicutes while increasing the abundance of Fusobacteria (Figure 7g). At the genus level, the abundance of *norank_f_norank_o_Chloroplast*, *Macrococcus*, *Leifsonia*, and *Aminobacter* significantly decreased in high-fat-fed carp, and the abundance of *Kaistia* increased, whereas L-carnitine significantly increased the abundance of *Cetobacterium* and *norank_f_norank_o_Chloroplast* (Figure 7h). The metabolic functions of the bacteria were then predicted, and it was found that the differential bacterial populations may affect energy production and conversion (Figure 7i).

LEfSe can be used to characterize the enriched microbiota (Figure S2). From the figure, it can be seen that the enrichment of the gut microbiota of carp in the control feed group was significantly higher than in the other two groups studied. Compared to the high-fat and L-carnitine groups, the intestinal flora of carp in the control group was significantly enriched with *Aminobacter*, *Lactobacillus*, *Macrococcus*, *Staphylococcus*, *Microbacteriaceae*, and *Brachybacterium*. The intestinal flora of high-fat-fed carp was significantly enriched with *Clostridium_sensu_stricto_13*, *Kaistia*, *Chitinophagales*, and *Acetitomaculum*. In contrast, the intestinal microorganism that was enriched in the L-carnitine group of carp was *Cetobacterium* of the *Fusobacteriaceae*.

### 3.9. L-Carnitine Enhanced Intestinal Metabolite Composition in High-Fat-Fed Carp

**Figure 8:**
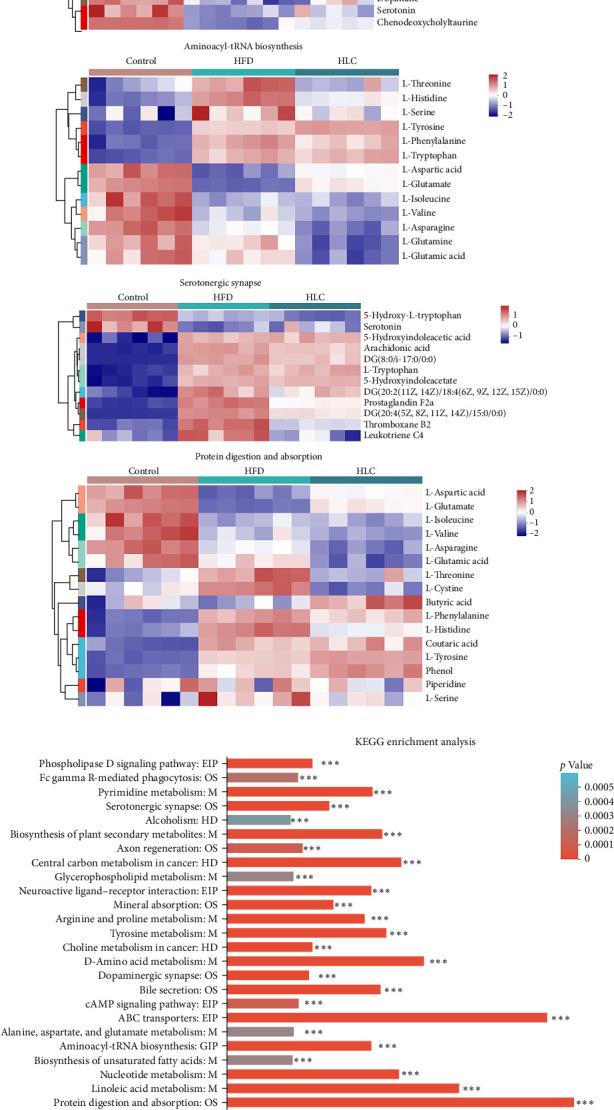
L-Carnitine influenced the metabolite composition and metabolic pathways of intestinal contents in high-fat-fed carp. (a) Heatmap of metabolite content in each differential pathway between different groups. (b) Differences between groups for different types of metabolites. *⁣*^*∗∗∗*^*p* < 0.001. HFD, high-fat diets; HLC, HFD supplemented with 1000 mg/kg L-carnitine.

By an untargeted metabolomics approach, 1689 differential metabolites were detected in the intestinal contents of three groups of carps (Figure S3a). The PLS-DA showed significant differences in metabolites among the three groups in both cationic and anionic models (Figure S3b). Further categorical analysis of the differential metabolites revealed that high-fat feeding upregulated the levels of 170 metabolites associated with lipid and lipid-like molecules, whereas L-carnitine downregulated the levels of 144 metabolites associated with lipid and lipid-like molecules (Figure S3c).  Figure 8a illustrates the changes in the levels of different metabolites in the protein digestion and absorption, bile secretion, 5-hydroxytryptaminergic synapses, and aminoacyl-tRNA biosynthesis pathways. Subsequently, the differential metabolites were analyzed for KEGG pathway enrichment (Figure 8b). Among them, the ABC transporter, linoleic acid metabolism, protein digestion and absorption, bile secretion, 5-hydroxytryptaminergic synapses, and aminoacyl-tRNA biosynthesis pathways were significantly enriched, suggesting that L-carnitine may regulate these pathways.

**Figure 9:**
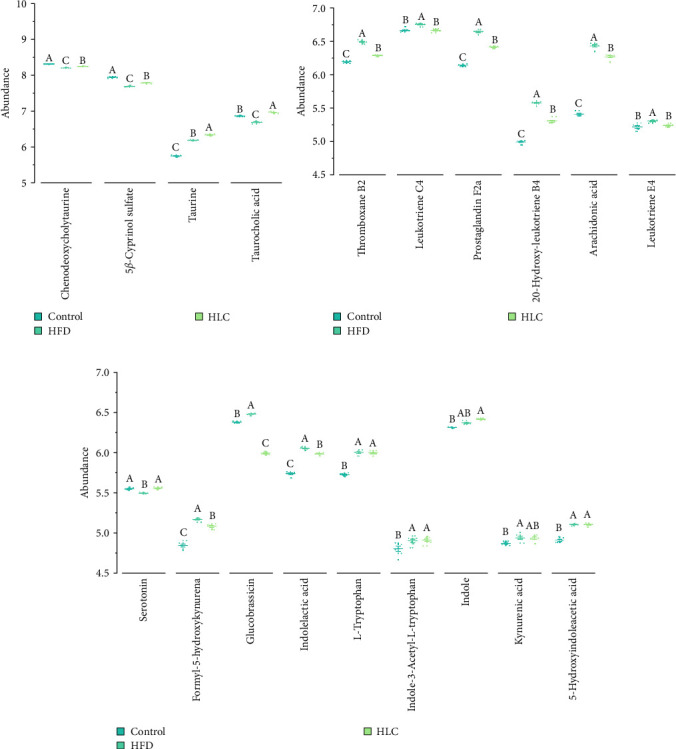
Influence of L-carnitine on the intestinal metabolite content of high-fat-fed carp. (a) Bile acid–related metabolites. (b) Arachidonic acid–related metabolites. (c) Tryptophan-related metabolites. Different letters indicate significant differences (*p* < 0.05). HFD, high-fat diets; HLC, HFD supplemented with 1000 mg/kg L-carnitine.

Figure 9 shows the content of bile acids, arachidonic acid, and tryptophan-related metabolites in the intestinal contents of different groups of carp. The results revealed that high-fat feeding decreased the contents of chenodeoxycholyltaurine, 5b-cyprinol sulfate, and taurocholic acid and significantly increased thromboxane B2, leukotriene C4, prostaglandin F2α, 20-hydroxy-leukotriene B4, arachidonic acid, leukotriene E4, glucobrassicin, and indolelactic acid. Whereas L-carnitine significantly increased the levels of chenodeoxycholyltaurine, 5b-cyprinol sulfate, taurine, taurocholic acid, and serotonin and significantly decreased thromboxane B2, leukotriene C4, prostaglandin F2α, 20-hydroxy-leukotriene B4, arachidonic acid, leukotriene E4, formyl-5-hydroxykynurena, glucobrassicin, and indolelactic acid for these metabolites (*p* < 0.05).

### 3.10. Correlation Analysis of Gut Flora Abundance With Gut Metabolite Content and Fish Health Indicators

**Figure 10:**
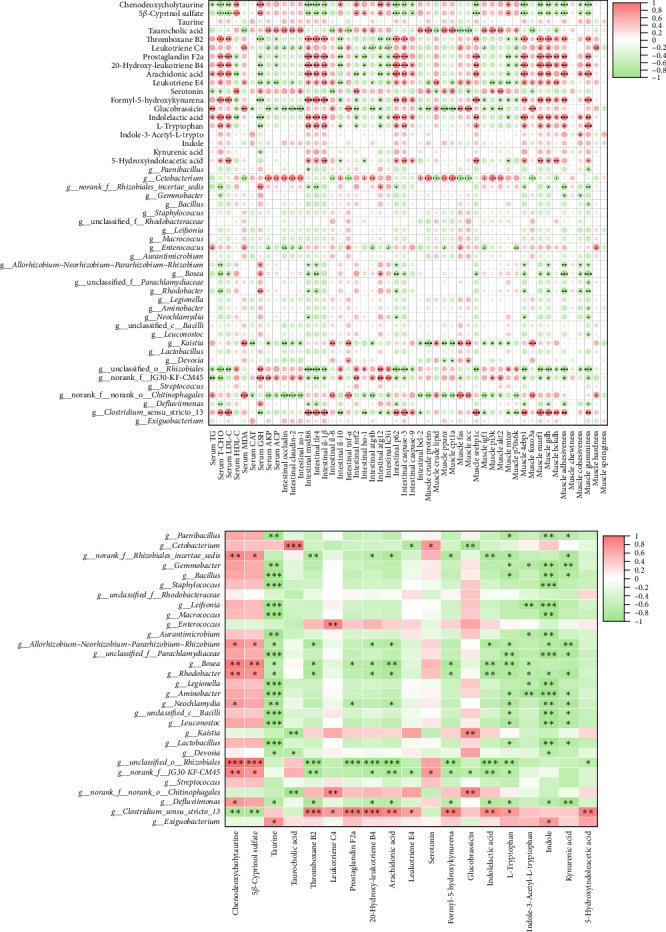
Analysis of the correlation of gut flora abundance with gut metabolite content and fish health indicators. (a) Spearman's correlation analysis of gut microorganisms and gut metabolites with fish health-related indicators. (b) Spearman correlation analysis of gut microbes and metabolites. The sizes of the circles in the correlation analysis chart represent the size of the correlation between the two indicators. The red color represents positive correlation, and the green color represents negative correlation. *⁣*^*∗*^ stands for *p* < 0.05, *⁣*^*∗∗*^ stands for *p* < 0.01, *⁣*^*∗∗∗*^ stands for *p* < 0.001.

Then, Spearman's correlation analysis of gut microorganisms, gut metabolites, and fish health-related indicators revealed that several genera and metabolites were significantly correlated with fish health-related indicators. Among them, *Cetobacterium*, *g_norank_f_norank_o_Chitinophagales*, taurocholic acid, glucobrassicin, and leukotriene E4 were significantly correlated with the enzymatic activities of ACP; AKP; tight junction-related genes; inflammation-related genes (*il-8*, *tnf-α*, and *il-10*); gene expression levels of *ho-1*, *atg4b*, and *bcl-2*; lipid metabolism-related genes (*pparα*, *cpt1a*, *fas*, and *acc*); protein metabolism-related genes (*igf-1*, *pi3k*, *akt2*, and *foxo3a*); and crude protein and crude lipid content positively or negatively (Figure 10a). The contents of thromboxane B2, prostaglandin F2α, arachidonic acid, and lLeukotriene E4 were significantly correlated with the levels of *caspase-3*, *caspase-9*, *myd88*, *tlr4*, *il-1β*, *4ebp1*, *murf-1*, *gdh*, and *bckdh*. Adhesiveness and gumminess were significantly positively correlated. *Bosea*, *Enterococcus*, *Rhodobacter*, and *Clostridium_sensu_stricto_13* were significantly correlated with muscle texture-related parameters (hardness, gumminess, and adhesiveness). The above findings demonstrate a correlation between the gut microbiota and gut metabolites with gut health, muscle nutrient metabolism, and muscle texture in carp, which suggests that the improvement of muscle quality and gut health by L-carnitine in high-fat-fed carp is related to the gut microbiota and gut metabolites.

To clarify the relationship between gut flora and metabolites, Spearman's correlation analysis was performed (Figure 10b). In the HLC group, increases in serotonin and dopamine were positively correlated with *Bosea* and *Rhodobacter* and negatively correlated with *Clostridium_sensu_stricto_13*. Likewise, decreases in leukotriene C4 and thromboxane B2 (TXB2) in the HLC group were negatively associated with *Gemmobacter* and *norank_f__JG30-KF-CM45* and positively associated with *Enterococcus* and *Clostridium_sensu_stricto_13*. Furthermore, *Cetobacterium* was significantly positively correlated with taurocholic acid and serotonin levels and significantly negatively correlated with leukotriene E4 content.

## 4. Discussion

L-Carnitine is a multipurpose nutrient that is shown to modulate lipid metabolism in the body, thereby mitigating the adverse effects of HFDs on animal growth, antioxidant performance, and lipid metabolism [5, 21]. Since less research has been conducted on the effects of L-carnitine on muscle nutrient metabolism and gut health in aquatic organisms, the present study investigated the effects of L-carnitine on muscle nutrient metabolism and intestinal health in high-fat-fed carp. Some studies have confirmed that L-carnitine supplementation improves the growth performance of gray mullet (*Mugil cephalus Linnaeus*) [22]. However, it was found that L-carnitine did not improve the growth performance of carp. This may be due to the fact that the growth-promoting effect of L-carnitine is also influenced by factors such as fish species, developmental stage, and diet composition [23]. In addition, the present study found that L-carnitine improved lipid levels, antioxidant capacity, and immune activity in high-fat-fed carp.

The muscle is the main edible part of fish, and muscle quality is an important assessment index of fish quality [24]. The evaluation of fish muscle quality includes muscle nutrient composition, muscle texture, and fatty acid composition [25]. High-fat feeding leads to disturbed muscle protein metabolism and altered muscle quality in aquatic organisms [7]. In the present study, L-carnitine was found to promote muscle lipolysis and protein anabolism to improve the morphology, muscle composition, and muscle texture of muscle fibers in high-fat-fed carp. Equally, L-carnitine alleviated the reduction in myofiber diameter induced by high-fat feeding of Nile tilapia [18] and increased the protein content of high-fat-fed juvenile largemouth bass [26]. In addition, myofiber density and diameter are important contributors to fish muscle texture [27]. Myofiber diameter as well as the composition and content of muscle nutrients significantly affects muscle quality [28]. Therefore, we hypothesized that L-carnitine may alter muscle nutrient composition by modulating muscle nutrient metabolism, influence muscle texture by increasing myofiber diameter, and ultimately affect muscle quality in high-fat-fed carp. In conclusion, L-carnitine improves the quality of the muscles in high-fat-fed carp.

The intestine is a key organ for immunity and digestion which is vital to the organismal health of aquatic organisms [29]. High-fat feeding can induce dysregulated expression of intestinal tight junction proteins, increased oxidative stress, heightened inflammatory response, compromised intestinal structure, and perturbed intestinal microbiota [30]. In this study, L-carnitine was found to increase the length of intestinal villi, reduce ntestinal inflammation and apoptosis, promote the expression of tight junction proteins and autophagy, protect the intestinal physical and mucosal barriers, and improve antioxidant capacity. It was found that decreased tight junction protein expression coincided with increased intestinal permeability [31]. Likewise, L-carnitine increased the level of tight junction proteins in the rooster duodenum [32]. Supplementation with dietary L-carnitine significantly reduced hepatopancreatic inflammatory response and improved antioxidant capacity in high-fat-fed juvenile black seabream [13]. L-Carnitine was similarly found to potentially alleviate oxidative and apoptotic processes in peripheral organs in a study in Wistar rats, decreasing caspase-3 activity and modulating Bcl-2 expression, thereby reducing levels of apoptosis [33]. In conclusion, L-carnitine improved intestinal health in high-fat fed carp.

Intestinal microbiota–host interactions are critical to animals health [34]. High-fat feeding can impact the body's metabolism by modifying the composition of intestinal flora, while targeting the gut microbiota can effectively adjust the body's metabolism [35]. Moreover, recent research has provided evidence that a HFD leads to a reduction in the diversity of gut microorganisms and an increase in the prevalence of the Firmicutes phylum [36]. Our investigation yielded similar results, showing an elevated abundance of the Firmicutes phylum with high-fat feeding, while supplementation with L-carnitine resulted in heightened microbial diversity, reduced Firmicutes abundance, and increased levels of the Fusobacteria phylum and *Cetobacterium* genus. *Cetobacterium* ameliorates gut microbiota disorders while improving hepatopancreatic health [37]. In addition, *Cetobacterium* can produce various amino acids such as isoleucine through intestinal metabolism [38]. In summary, L-carnitine can alleviate the gut microbiota disorders in carp caused by high-fat feeding.

Gut metabolites are critical in gut microbe–host interactions [39]. Therefore, we examined the composition of intestinal metabolites and found that L-carnitine significantly raised intestinal bile acid and serotonin levels and significantly lowered arachidonic acid and its metabolites. Taurine, a bile acid, attenuates inflammation and oxidative stress-mediated damage in certain diseases and has a broad protective effect against bacterial infections in several fish species while increasing fish survival [40]. Serotonin is essential for host intestinal health and systemic homeostasis by enhancing nutrient absorption and storage and regulating the composition of the gut microbiota, thereby promoting the colonization of beneficial bacteria in the gut flora and inhibiting pathogenic bacteria [41, 42]. Excessive conversion of linoleic acid to arachidonic acid is a major factor in inducing inflammation and obesity [43]. Arachidonic acid itself is an intracellular signaling molecule closely associated with proinflammatory microbiota. The metabolites of arachidonic acid are mainly prostaglandins, thromboxanes, and leukotrienes, which may be involved in inducing inflammation [44, 45]. Correlation analyses showed that the *Cetobacterium* genus, considered a beneficial genus, was positively correlated with gut compactness and significantly and negatively correlated with oxidative stress, inflammatory response, and lipid synthesis. *Cetobacterium* is a beneficial anaerobic bacterium that increases the diversity of the intestinal flora and promotes intestinal health in fish through the secretion of metabolites [46]. *Cetobacterium* and several other genera have also been found to be significantly correlated with the metabolism of nutrients in muscle, muscle composition, and indices related to muscle texture. It has been suggested that gut microbiota composition may determine skeletal muscle metabolism and function [47]. Therefore, we hypothesized that L-carnitine may improve intestinal health in high-fat-fed carp by modulating gut microbiota and intestinal metabolites. Furthermore, this study found that the modulatory effect of L-carnitine on muscle quality in high-fat-fed carp was closely related to altering intestinal flora, but the modulatory mechanism requires further investigation.

## 5. Conclusion

The present study showed that the addition of 500 mg/kg and 1000 mg/kg of L-carnitine to HFDs had a positive effect on alleviating a number of adverse physiological effects induced by high-fat feeding. First, L-carnitine could effectively reduce the dyslipidaemia of carp caused by high-fat feeding. In addition, it can improve the efficiency of the body's utilization of lipids by regulating muscle nutrient metabolism, which promotes the deposition of proteins in the muscle, thus affecting muscle fiber morphology and muscle composition, and has an effect on muscle texture. L-Carnitine also has a positive effect on the intestines of carp. It not only alleviates the morphological damage to the intestinal tissue caused by a HFD but also reduces the level of inflammation in the intestine. In addition, L-carnitine reduces intestinal permeability by increasing the expression of genes related to intestinal tight junction proteins, effectively protecting the physical barrier of the intestinal tract. In addition, L-carnitine played a positive role in increasing the intestinal microbial diversity and altering its metabolite composition in high-fat fed carp, which helped to alleviate the intestinal flora imbalance induced by high-fat feeding and maintain the stability of the intestinal microecology. Furthermore, L-carnitine regulates muscle nutrient metabolism and gut health in relation to gut microbiota. This study provides a reference for the mechanism of action of L-carnitine and its practical application.

## Figures and Tables

**Table 1:** Fatty acid composition of different dietary carps.

Ingredient	Control	HFD	LLC	HLC
Fine rice bran	125	125	125	125
Soybean meal	300	300	300	300
Rapeseed meal	100	100	100	100
Cottonseed meal	180	180	180	180
Fish meal	40	40	40	40
Wheat	80	80	80	80
Microcrystalline cellulose	80	20	19.5	19
Ca(H_2_PO_4_)_2_	20	20	20	20
Zeolite powder	10	10	10	10
Bentonite	10	10	10	10
Premix material^†^	10	10	10	10
Bean oil	45	105	105	105
L-Carnitine	0	0	0.5	1
Total	1000	1000	1000	1000
Proximate composition (%)^‡^	—	—	—	—
Moisture	8.87 ± 0.51	7.79 ± 0.10	8.30 ± 0.23	8.00 ± 0.33
Crude protein	32.29 ± 0.86	31.47 ± 0.74	31.69 ± 0.87	31.40 ± 0.73
Crude lipid	6.21 ± 0.31^b^	11.20 ± 0.90^a^	11.40 ± 0.52^a^	11.31 ± 0.16^a^

*Note:* All values are presented as mean ± SEM (*n* = 6). Different letters indicate significant differences (*p* < 0.05).

Abbreviations: HFD, high-fat diet; HLC, HFD supplemented with 1000 mg/kg L-carnitine; LLC, HFD supplemented with 500 mg/kg L-carnitine.

^†^The premix provided the following per kg of diets: Zn 25 mg, Cu 3 mg, Fe 125 mg, Mn 15 mg, I 0.6 mg, Co 0.1 mg, Se 0.4 mg, vitamin A 8000 IU, vitamin C 500 mg, vitamin D3 3000 IU, vitamin E 60 mg, vitamin K3 5 mg, vitamin B2 30 mg, vitamin B6 15 mg, vitamin B12 0.5 mg, nicotinamide 175 mg, pantothenate acid 50 mg, folic acid 5 mg, biotin 2.5 mg, Inositol 1000 mg.

^‡^The nutrient levels were measured values.

**Table 2:** Impact of L-carnitine on the growth performance of high-fat-fed carp.

Parameters	Control	HFD	LLC	HLC
Initial body weight (g)	16.53 ± 0.41	16.20 ± 0.50	16.48 ± 0.43	16.54 ± 0.42
Survival rate (%)	100	100	100	100
Final body weight (g)	39.78 ± 0.42^b^	41.85 ± 0.43^a^	40.44 ± 0.42^a,b^	40.39 ± 0.40^a,b^
Weight gain rate (%)	140.73 ± 2.36^b^	158.56 ± 4.51^a^	145.35 ± 2.03^a,b^	144.38 ± 4.45^a,b^
Specific growth rate (%/day)	1.57 ± 0.02	1.70 ± 0.05	1.60 ± 0.01	1.60 ± 0.03
Hepatosomatic index (%)	0.87 ± 0.03	1.05 ± 0.04	0.94 ± 0.07	0.81 ± 0.07
Viscerosomatic index (%)	6.39 ± 0.34	6.82 ± 0.26	6.77 ± 0.35	5.57 ± 0.31
Feed efficiency ratio (%)	1.61 ± 0.03	1.56 ± 0.04	1.66 ± 0.01	1.63 ± 0.03

*Note*: Different letters indicate significant differences (*p* < 0.05).

Abbreviations: HFD, high-fat diet; HLC, HFD supplemented with 1000 mg/kg L-carnitine; LLC, HFD supplemented with 500 mg/kg L-carnitine.

**Table 3:** L-Carnitine influenced fatty acid composition in high-fat-fed carp.

Item	Control	HFD	LLC	HLC
C14:0	0.30 ± 0.02	0.28 ± 0.04	0.23 ± 0.03	0.30 ± 0.06
C15:0	0.21 ± 0.00	0.22 ± 0.03	0.20 ± 0.00	0.19 ± 0.02
C16:0	19.08 ± 0.29^a^	17.01 ± 0.53^b^	16.69 ± 0.19^b^	17.03 ± 0.25^b^
C17:0	9.32 ± 0.71^a^	4.37 ± 1.34^b^	0.13 ± 0.06^c^	0.25 ± 0.01^c^
C18:0	7.67 ± 0.23^b^	7.49 ± 0.22^b^	8.64 ± 0.54^a^	7.81 ± 0.14^a,b^
C16:1	2.34 ± 0.13^a^	1.73 ± 0.12^b,c^	1.56 ± 0.06^c^	2.09 ± 0.25^a,b^
C17:1	0.30 ± 0.00	0.27 ± 0.02	0.27 ± 0.06	0.29 ± 0.03
C18:1	20.26 ± 0.62^a,b^	20.13 ± 0.81^a,b^	19.09 ± 0.65^b^	23.68 ± 1.82^a^
C20:1	2.60 ± 0.29^a^	1.75 ± 0.09^b^	1.57 ± 0.16^b^	2.20 ± 0.42^a,b^
C20:2	1.23 ± 0.06^a^	0.97 ± 0.04^c^	1.04 ± 0.03^b,c^	1.11 ± 0.02^b^
C18:2n-6	18.20 ± 0.39^c^	30.94 ± 1.53^a^	33.59 ± 0.56^a^	25.64 ± 2.01^b^
C18:3n-3	0.93 ± 0.03^c^	2.35 ± 0.16^a^	2.39 ± 0.09^a^	1.71 ± 0.21^b^
C18:3n-6	0.45 ± 0.12	0.60 ± 0.19	0.45 ± 0.01	0.54 ± 0.19
C20:3n-3	6.92 ± 0.37^a^	4.64 ± 0.22^c^	5.37 ± 0.23^b^	6.04 ± 0.19^b^
C20:3n-6	2.75 ± 0.13^a^	2.32 ± 0.10^b^	2.73 ± 0.07^a^	2.55 ± 0.07^a,b^
C20:5n-3	1.17 ± 0.04^a^	0.66 ± 0.05^c^	0.80 ± 0.01^b^	1.17 ± 0.01^a^
C22:6n-3	6.27 ± 0.19^a^	4.26 ± 0.32^c^	5.23 ± 0.14^b^	5.62 ± 0.19^b^
∑SA	36.58 ± 0.72^a^	29.37 ± 2.92^b^	25.89 ± 0.64^b^	28.40 ± 4.43^b^
∑MUFA	25.49 ± 0.39^a,b^	23.88 ± 0.48^a,b^	22.49 ± 0.28^b^	27.21 ± 1.45^a^
∑PUFA	37.93 ± 0.32^c^	46.75 ± 2.19^b^	51.61 ± 0.18^a^	44.39 ± 1.91^b^
∑SA/∑PUFA	0.96 ± 0.02^b^	0.63 ± 0.09^c^	0.50 ± 0.01^c^	0.64 ± 0.21^a^
∑n-3 PUFA	15.29 ± 0.55^a^	11.90 ± 0.68^c^	13.80 ± 0.29^b^	14.54 ± 0.16^a,b^
∑n-6 PUFA	21.41 ± 0.34^c^	33.87 ± 1.63^a^	36.77 ± 0.50^a^	28.73 ± 2.07^b^
n-3/n-6PUFA	0.71 ± 0.04^b^	0.35 ± 0.02^c^	0.38 ± 0.01^c^	0.51 ± 0.04^a^

*Note:* All values are presented as mean ± SEM (*n* = 6). Different letters indicate significant differences (*p* < 0.05).

Abbreviations: HFD, high-fat diet; HLC, HFD supplemented with 1000 mg/kg L-carnitine; LLC, HFD supplemented with 500 mg/kg L-carnitine.

## Data Availability

The data that support the findings of this study are available from the corresponding author upon reasonable request.

## Nomenclature

HFD: High-fat diet
TXB2: Thromboxane B2
PCA: Principal component analysis
CPT-I: Carnitine palmitoyltransferase I
CACT: Carnitine acylcarnitine translocase.

## Supporting Information

Supporting Information The supporting information include Table S1, Figure S1, Figure S2, and Figure S3. The supporting information of this manuscript describe the primer sequences mentioned in the manuscript and some experimental results.

## References

[1] Zhang M., Wang S., Gan L. (2021). Effects of Fishmeal Replacement With Eight Protein Sources on Growth Performance, Blood Biochemistry and Stress Resistance in *Opsariichthys bidens*. *Aquaculture Nutrition*.

[2] Wang J., Mai K., Ai Q. (2022). Conventional Soybean Meal as Fishmeal Alternative in Diets of Japanese Seabass (*Lateolabrax japonicus*): Effects of Functional Additives on Growth, Immunity, Antioxidant Capacity and Disease Resistance. *Antioxidants*.

[3] Sabzi E., Mohammadiazarm H., Salati A. P. (2017). Effect of Dietary L-Carnitine and Lipid Levels on Growth Performance, Blood Biochemical Parameters and Antioxidant Status in Juvenile Common Carp (*Cyprinus carpio*). *Aquaculture*.

[4] Pohlenz C., Gatlin D. M. (2014). Interrelationships Between Fish Nutrition and Health. *Aquaculture*.

[5] Li J. M., Li L. Y., Qin X. (2017). Systemic Regulation of L-Carnitine in Nutritional Metabolism in Zebrafish, *Danio rerio*. *Scientific Reports*.

[6] Wang C., Zhang C., Yu H. B. (2022). Glycerol Monolaurate and Triglycerol Monolaurate Alleviated High-Fat Diet Induced Lipid Accumulation and Damage of Liver in Zebrafish (*Danio rerio*). *Aquaculture*.

[7] Zhou W. H., Limbu S. M., Li R. X. (2023). Dietary Sodium Acetate Improves High-Fat Diet Utilization Through Promoting Differential Nutrients Metabolism Between Liver and Muscle in Nile Tilapia (*Oreochromis niloticus*). *Aquaculture*.

[8] Arias-Jayo N., Abecia L., Alonso-Sáez L., Ramirez-Garcia A., Rodriguez A., Pardo M. A. (2018). High-Fat Diet Consumption Induces Microbiota Dysbiosis and Intestinal Inflammation in Zebrafish. *Microbial Ecology*.

[9] Hu B. F., Ye C., Leung E. L. (2020). *Bletilla striata* Oligosaccharides Improve Metabolic Syndrome Through Modulation of Gut Microbiota and Intestinal Metabolites in High Fat Diet-Fed Mice. *Pharmacological Research*.

[10] Carillo M. R., Bertapelle C., Scialò F. (2020). L-Carnitine in Drosophila: A Review. *Antioxidants*.

[11] Liu L., Long X. W., Deng D., Cheng Y. X., Wu X. G. (2018). Molecular Characterization and Tissue Distribution of Carnitine Palmitoyltransferases in Chinese Mitten Crab *Eriocheir sinensis* and the Effect of Dietary Fish Oil Replacement on Their Expression in the Hepatopancreas. *PLOS ONE*.

[12] Haghighatdoost F., Jabbari M., Hariri M. (2019). The Effect of L-Carnitine on Inflammatory Mediators: A Systematic Review and Meta-Analysis of Randomized Clinical Trials. *European Journal of Clinical Pharmacology*.

[13] Jin M., Pan T., Cheng X. (2019). Effects of Supplemental Dietary L-Carnitine and Bile Acids on Growth Performance, Antioxidant and Immune Ability, Histopathological Changes and Inflammatory Response in Juvenile Black Seabream (*Acanthopagrus schlegelii*) Fed High-Fat Diet. *Aquaculture*.

[14] Dos Santos Sanchez M. S., Lins-Rodrigues M., Pessini J. E., Bittencourt F., Boscolo W. R., Signor A. (2021). Dietary Supplementation With l-Carnitine for Nile Tilapia Juveniles. *Aquaculture*.

[15] Wang J. G., Rahimnejad S., Liu Y.-C. (2022). Dietary L-Carnitine Supplementation Affects Flesh Quality Through Modifying the Nutritional Value and Myofibers Morphological Characteristics in Largemouth Bass (*Micropterus salmoides*). *Animal Feed Science and Technology*.

[16] Zhang Y., Gao K., Ren Y. (2022). Broken Xinyang Maojian Tea Supplementation in a High-Fat Diet Improves the Growth Performance, Flesh Quality and Lipid Metabolism of Yellow River Carp (*Cyprinus carpio*). *Aquaculture Reports*.

[17] AOAC (2000). *Official Methods of Analysis of AOAC International*.

[18] Zhang Z. Y., Limbu S. M., Zhao S. H. (2022). Dietary l-Carnitine Supplementation Recovers the Increased pH and Hardness in Fillets Caused by High-Fat Diet in Nile Tilapia (*Oreochromis niloticus*). *Food Chemistry*.

[19] Peng M., Xu W., Mai K. (2014). Growth Performance, Lipid Deposition and Hepatic Lipid Metabolism Related Gene Expression in Juvenile Turbot (*Scophthalmus maximus L*.) Fed Diets With Various Fish Oil Substitution Levels by Soybean Oil. *Aquaculture*.

[20] Livak K. J., Schmittgen T. D. (2001). Analysis of Relative Gene Expression Data Using Real-Time Quantitative PCR and the 2−*ΔΔ*CT Method. *Methods*.

[21] Gao X., Sun C., Zhang Y., Hu S., Li D. (2022). Dietary Supplementation of l-Carnitine Ameliorates Metabolic Syndrome Independent of Trimethylamine N-Oxide Produced by Gut Microbes in High-Fat Diet-Induced Obese Mice. *Food & Function*.

[22] Akbary P. (2019). Growth Performance, Biochemical Indices and Antioxidant Status of Grey Mullet (*Mugil cephalus Linnaeus*, 1758) Under Dietary L-Carnitine Supplementation. *Proceedings of the National Academy of Sciences, India Section B: Biological Sciences*.

[23] Mohseni M., Ozorio R. O. A., Pourkazemi M., Bai S. C. (2008). Effects of Dietary l-Carnitine Supplements on Growth and Body Composition in Beluga Sturgeon (*Huso huso*) Juveniles. *Journal of Applied Ichthyology*.

[24] Zhang T., Zhang L., Yin T. (2023). Recent Understanding of Stress Response on Muscle Quality of Fish: From the Perspective of Industrial Chain. *Trends in Food Science & Technology*.

[25] Jiang W. D., Chen L., Liu Y. (2019). Impact and Consequences of Dietary Riboflavin Deficiency Treatment on Flesh Quality Loss in on-Growing Grass Carp (*Ctenopharyngodon idella*). *Food & Function*.

[26] Liu Y. C., Limbu S. M., Wang J. G. (2022). Dietary L-Carnitine Alleviates the Adverse Effects Caused by Reducing Protein and Increasing Fat Contents in Diet Juvenile Largemouth Bass (*Micropterus salmoides*). *Aquaculture Nutrition*.

[27] Castro P. L., Rincón L., Álvarez B., Ginés R. (2021). Texture Changes During Chilled Storage of Wild and Farmed Blackspot Seabream (*Pagellus bogaraveo*) Fed Different Diets. *Food Science & Nutrition*.

[28] Zhang Z. Y., Jiang Z. Y., Lv H. B. (2021). Dietary Aflatoxin Impairs Flesh Quality Through Reducing Nutritional Value and Changing Myofiber Characteristics in Yellow Catfish (*Pelteobagrus Fulvidraco*). *Animal Feed Science and Technology*.

[29] Holtof M., Lenaerts C., Cullen D., Broeck J. V. (2019). Extracellular Nutrient Digestion and Absorption in the Insect Gut. *Cell and Tissue Research*.

[30] Okyere S. K., Wen J., Cui Y. (2022). *Bacillus toyonensis* SAU-19 and SAU-20 Isolated From, *Ageratina adenophora*, Alleviates the Intestinal Structure and Integrity Damage Associated With Gut Dysbiosis in Mice Fed High Fat Diet. *Frontiers in Microbiology*.

[31] Zhan L., Pu J., Zheng J. (2022). *Tetrastigma hemsleyanum*, Diels et Gilg Ameliorates Lipopolysaccharide Induced Sepsis via Repairing the Intestinal Mucosal Barrier. *Biomedicine & Pharmacotherapy*.

[32] Ennab W., Ye N., Wu H. (2023). The Synergistic Effects of the Combination of L-Carnitine and Lycopene on the Lycopene Bioavailability and Duodenal Health of Roosters. *Animals*.

[33] Kelek S. E., Afşar E., Akçay G., Danışman B., Aslan M. (2019). Effect of Chronic L-Carnitine Supplementation on Carnitine Levels, Oxidative Stress and Apoptotic Markers in Peripheral Organs of Adult Wistar Rats. *Food and Chemical Toxicology*.

[34] Liu H., Guo X. W., Gooneratne R. (2016). The Gut Microbiome and Degradation Enzyme Activity of Wild Freshwater Fishes Influenced by Their Trophic Levels. *Scientific Reports*.

[35] Guo C., Han L., Li M., Yu L. (2020). Seabuckthorn (*Hippophaë Rhamnoides*) Freeze-Dried Powder Protects Against High-Fat Diet-Induced Obesity, Lipid Metabolism Disorders by Modulating the Gut Microbiota of Mice. *Nutrients*.

[36] Wang L., Sagada G., Xu B., Zhang J., Shao Q. (2022). Influence of Dietary Berberine on Liver Immune Response and Intestinal Health of Black Sea Bream (*Acanthopagrus schlegelii*) Fed With Normal and High-Lipid Diets. *Aquaculture Nutrition*.

[37] Zhao Y., Li S., Lessing D. J., Chu W. (2024). The Attenuating Effects of Synbiotic Containing *Cetobacterium* Somerae and Astragalus Polysaccharide Against Trichlorfon-Induced Hepatotoxicity in Crucian Carp (*Carassius carassius*). *Journal of Hazardous Materials*.

[38] Xie M., Zhou W., Xie Y. (2021). Effects of *Cetobacterium* Somerae Fermentation Product on Gut and Liver Health of Common Carp (*Cyprinus carpio*) Fed Diet Supplemented With Ultra-Micro Ground Mixed Plant Proteins. *Aquaculture*.

[39] Gan G. W., Lin S. H., Luo Y. F. (2024). Unveiling the Oral-Gut Connection: Chronic Apical Periodontitis Accelerates Atherosclerosis via Gut Microbiota Dysbiosis and Altered Metabolites in apoE−/− Mice on a High-Fat Diet. *International Journal of Oral Science*.

[40] Mastrella L., Moretti P., Pieraccini S., Magi S., Piccirillo S., Ortore M. G. (2022). Taurine Stabilizing Effect on Lysozyme. *Life*.

[41] Akram N., Faisal Z., Irfan R. (2024). Exploring the Serotonin-Probiotics-Gut Health Axis: A Review of Current Evidence and Potential Mechanisms. *Food Science & Nutrition*.

[42] Su X., Gao Y., Yang R. (2022). Gut Microbiota-Derived Tryptophan Metabolites Maintain Gut and Systemic Homeostasis. *Cells*.

[43] Naughton S. S., Mathai M. L., Hryciw D. H., McAinch A. J. (2016). Linoleic Acid and the Pathogenesis of Obesity. *Prostaglandins & Other Lipid Mediators*.

[44] Zhuang K., Shu X., Meng W., Zhang D. (2024). Blended-Protein Changes Body Weight Gain and Intestinal Tissue Morphology in Rats by Regulating Arachidonic Acid Metabolism and Secondary Bile Acid Biosynthesis Induced by Gut Microbiota. *European Journal of Nutrition*.

[45] Ortiz-Placín C., Castillejo-Rufo A., Estarás M., González A. (2023). Membrane Lipid Derivatives: Roles of Arachidonic Acid and Its Metabolites in Pancreatic Physiology and Pathophysiology. *Molecules*.

[46] Shi Y., Liu Y., Xie K. (2023). Sanguinarine Improves Intestinal Health in Grass Carp Fed High-Fat Diets: Involvement of Antioxidant, Physical and Immune Barrier, and Intestinal Microbiota. *Antioxidants*.

[47] Giron M., Thomas M., Dardevet D., Chassard C., Savary-Auzeloux I. (2022). Gut Microbes and Muscle Function: Can Probiotics Make Our Muscles Stronger?. *Journal of Cachexia, Sarcopenia and Muscle*.

