# Supplementary material for: L-Carnitine Improves Muscle Nutrient Metabolism and Intestinal Health in High-Fat-Fed Carp (Cyprinus carpio)
Source: Aquac Nutr. 2025 Jan 9;2025:5623889. doi: 10.1155/anu/5623889 (PMC11737909; doi:10.1155/anu/5623889)
Supplement: Supporting Information — The supporting information include Table S1, Figure S1, Figure S2, and Figure S3. The supporting information of this manuscript describe the primer sequences mentioned in the manuscript and some experimental results. [file 5623889.f1.docx]

**Supplementary Materials**

**L-carnitine improves muscle nutrient metabolism and intestinal health in high-fat-fed carp (*Cyprinus carpio*)**

Xianglin Cao^1^, Rongjie Yuan^1, 2^, Yi Guo^2^, Mengtao Jia^2^, Yinyin Wei^2^, Jiameng zhou^2^, Han Cui^1^, Baohua Li^1^, Jianjun Chen^2^

^1^ College of Fisheries, Henan Normal University, Xinxiang 453007, China

^2^ College of Life Science, Henan Normal University, Xinxiang 453007, China

Corresponding author: Jianjun Chen, E-mail: [chenjianjun@htu.edu.cn](mailto:chenjianjun@htu.edu.cn);

Address: College of Life Science, Henan Normal University, Xinxiang, 453007, China

ORCID: 0000-0002-8050-5900

**Table S1** Primer sequences.

| **Primer** | **Forward** | **Reverse** | **Acc. Num.** |
| --- | --- | --- | --- |
| *pparα* | GTGCGAGAGCATAGTTGGCTT | TGGCAGAGTGCTCGAGTTG | [XM_042722106.1](https://www.ncbi.nlm.nih.gov/nuccore/XM_042722106.1) |
| *lpl* | CGCTCCATTCACCTGTTCAT | GCTGAGACACATGCCCTTATT | XM_042712447.1 |
| *cpt-1a* | CAGATGGAAAGTGTTGCTAATGAC | TGTGTAGAAGTTGCTGTTGACCA | XM_019122018.1 |
| *fas* | GAGAAAGTGGTGGTCGCTAAA | CATCACGCACACCCAGAATA | XM_019070657.1 |
| *acc* | TTCACTGGCGTATGAGGATATC | TCCACCTGTATGGTTCTTTGG | [XM_042723589.1](https://www.ncbi.nlm.nih.gov/nuccore/XM_042723589.1) |
| *srebp-1c* | CGTCTGCTTCACTTCACTACTC | GGACCAGTCTTCATCCACAAA | XM_019073034.1 |
| *igf-1* | GGCGTAAACCCACACAACTG | GTGGAACGGACGAACATCAC | XM_019092966.2 |
| *pi3k* | ACCTGCCGATGCTTCTGAAACG | TCCTCTGGCTTCTCCTGCTTGG | XM_042750094.1 |
| *akt-2* | CGGCGTAAACCCACACAAC | ACGTGGAACGGACGAACAT | XM_019100085.2 |
| *mtor* | TGCGGAGTATGTGGAGTT | CATCTCTTTGGTCTCTCTCTGG | XM_019108641.1 |
| *p70s6k* | GGGAGAAGAAAGGTCCCCAC | \| AATGCGCTCTGAGGTTTTGG \| \| --- \| | XM_042771062.1 |
| *4ebp1* | GCTACCTCACGACTATTGC | TTCTTGCTTGTCACTCCTG | XM_019077307.2 |
| *foxo3a* | CGGCGTAAACCCACACAAC | ACGTGGAACGGACGAACAT | XM_019121026.2 |
| *murf-1* | CAAACAGCAGCAGGAAGGTG | CGATGGCGTTACTCAGCTCT | XM_042751511.1 |
| *gdh* | ACTATTGCCCACACCGAT | CAGACCCACATTACCGAA | XM_042767684.1 |
| *bckdh* | CGGCTCCATCTATCACTCCC | ACCTGCTCCACTGCTGCTCT | XM_042740427.1 |
| *claudin-2* | CTGGAGTTGATGGGTTTCTTTTG | AGACCTTTCATGCTTTCTACCG | KU200691.1 |
| *occludin* | GACGCCATGGATGAGTACAA | GTGGTTGAGTTTGGCTTTCAG | KF975606.1 |
| *zonula occludens-1* | GATATGTTCGGAGGTGCGCT | ATGTTGCATGGTGCTTGCTG | KY290394.1 |
| *nrf-2* | GTATGACCCAGAGACAAACC | CCAACCACATACAGACTTCC | XM_019124298.1 |
| *ho-1* | GCTCATGCATATACGCGCTA | CAGAATTCCCTTGTTGCCACT | XM_019071460.1 |
| *caspase-3* | TCGCAGGACAGGCATGAAC | CACTAACGAAGCACAGCGG | XM_019110173.1 |
| *caspase-9* | TGATCCCTCTGCCAGTCC | TCTCCATCTTGTCGCAGT | XM_019066459.1 |
| *bcl-2* | ATGTGCGTGGAAAGCGTCAAC | AAAGGCTCCGATGGTCACTCC | KJ174686 |
| *atg-12* | ACAGTACAGTCACTCGCTCA | AAAACACTCGAAAAGCACACC | XM_019125508.1 |
| *atg-4b* | GTAATAGGTGGGAAGCCGAACAGTG | AGTGGTAGGAGTCGTCTGGGAAC | XM_042725815.1 |
| *p62* | AAGACCAAGGCAGTGATGAGGAATG | GCTTGTGCTGGAGTCGGTACTTAG | XM_042737795.1 |
| *lc3ii* | GCCATCCGACAGACCCTT | TGAGCTGCAGTCTACGCC | XM_019072575.1 |
| *il-10* | CAGCGCATCTTGAGTGTTTAC | TCATGACGTGACAGCCATAAG | AB110780 |
| *tnf-α* | AGCCAGGTGTCTTTCCACAT | ATGTAGCCGCCATAGGAATCG | XM_019088899.1 |
| *il-1β* | CAAACTGGAGCTGTCTTCGC | CTTCACCAGACGCTCTTCGAT | AB010701.1 |
| *il-8* | TGTTGCTCTGCACAAAACTGG | ATGCATTCGTGGGCACCT | XM_019080796.2 |
| *myd88* | CGCCGAAATGATGGACTTCAC | TCTACTGTTGCCTCTGGACG | HQ380208.1 |
| *tlr4* | CGAGTCAGAACCTCAGCC | CCTTGCTCCCCCTCATCC | XM_019090185.1 |
| *40s* | CCGTGGGTGACATCGTTACA | TCAGGACATTGAACCTCACTGTCT | XM_019078334 |


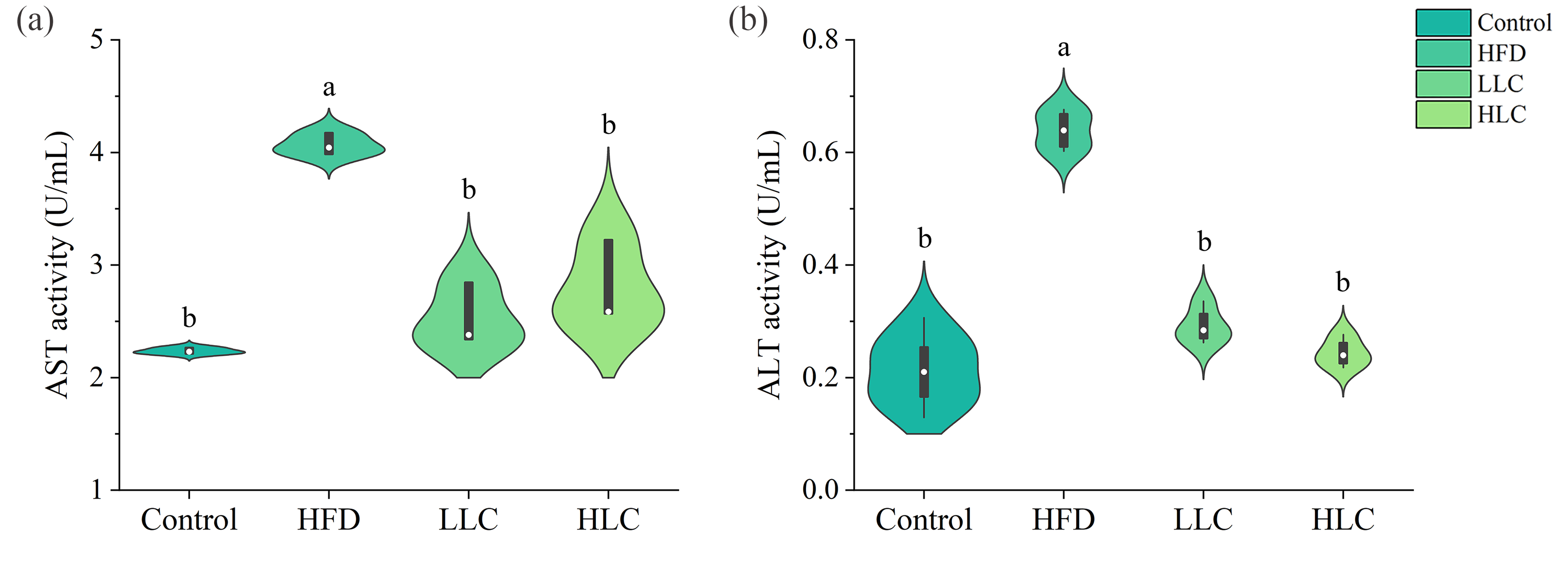


**Figure S1.** The influence of L-carnitine on serum biochemical indices of high-fat-fed carp. (a) Serum AST activity; (b) ALT activity of Serum;


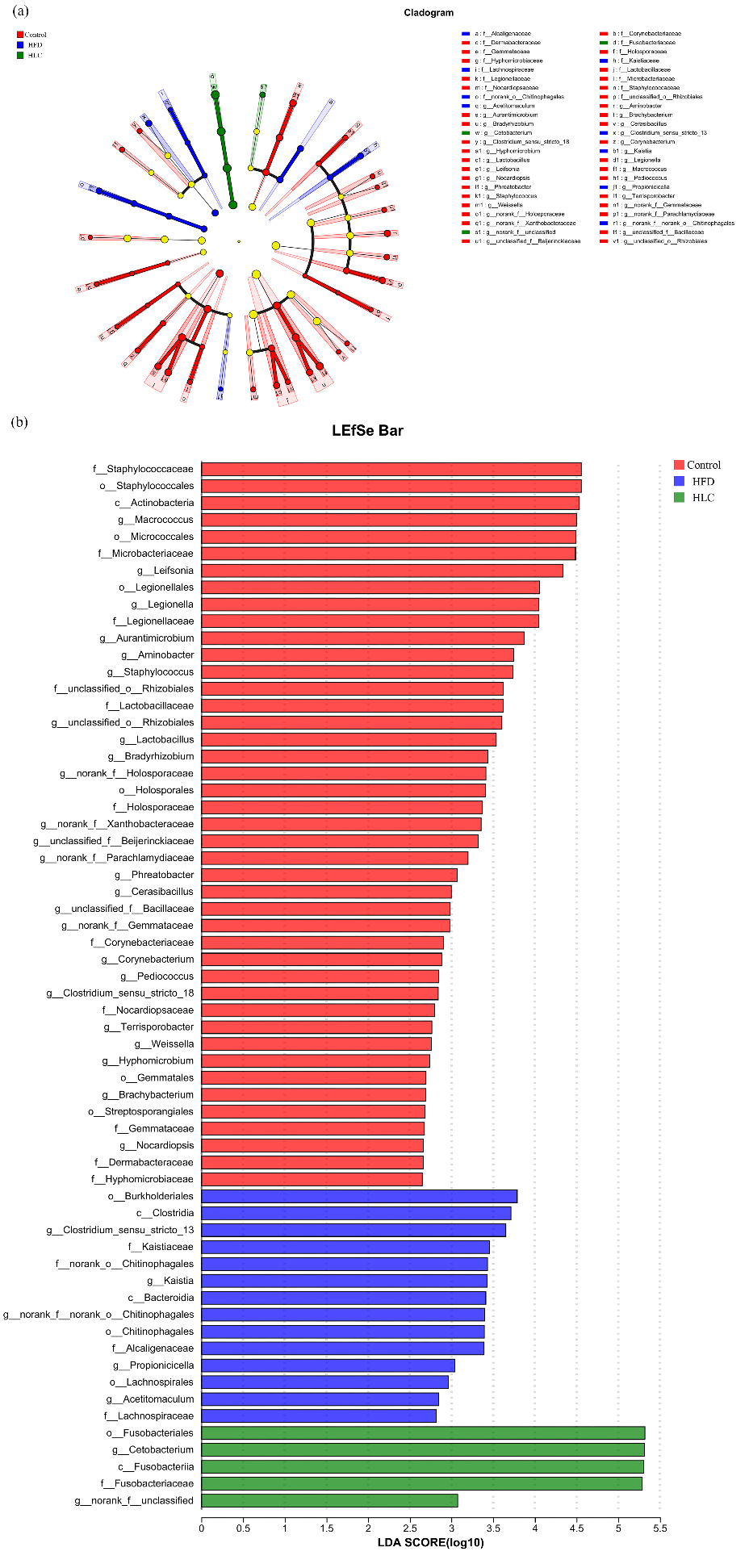


**Figure S2.** LEfSe analysis of significantly enriched communities of microorganisms. (a) Evolutionary branching diagram; (b) Histogram of the distribution of LDA values.


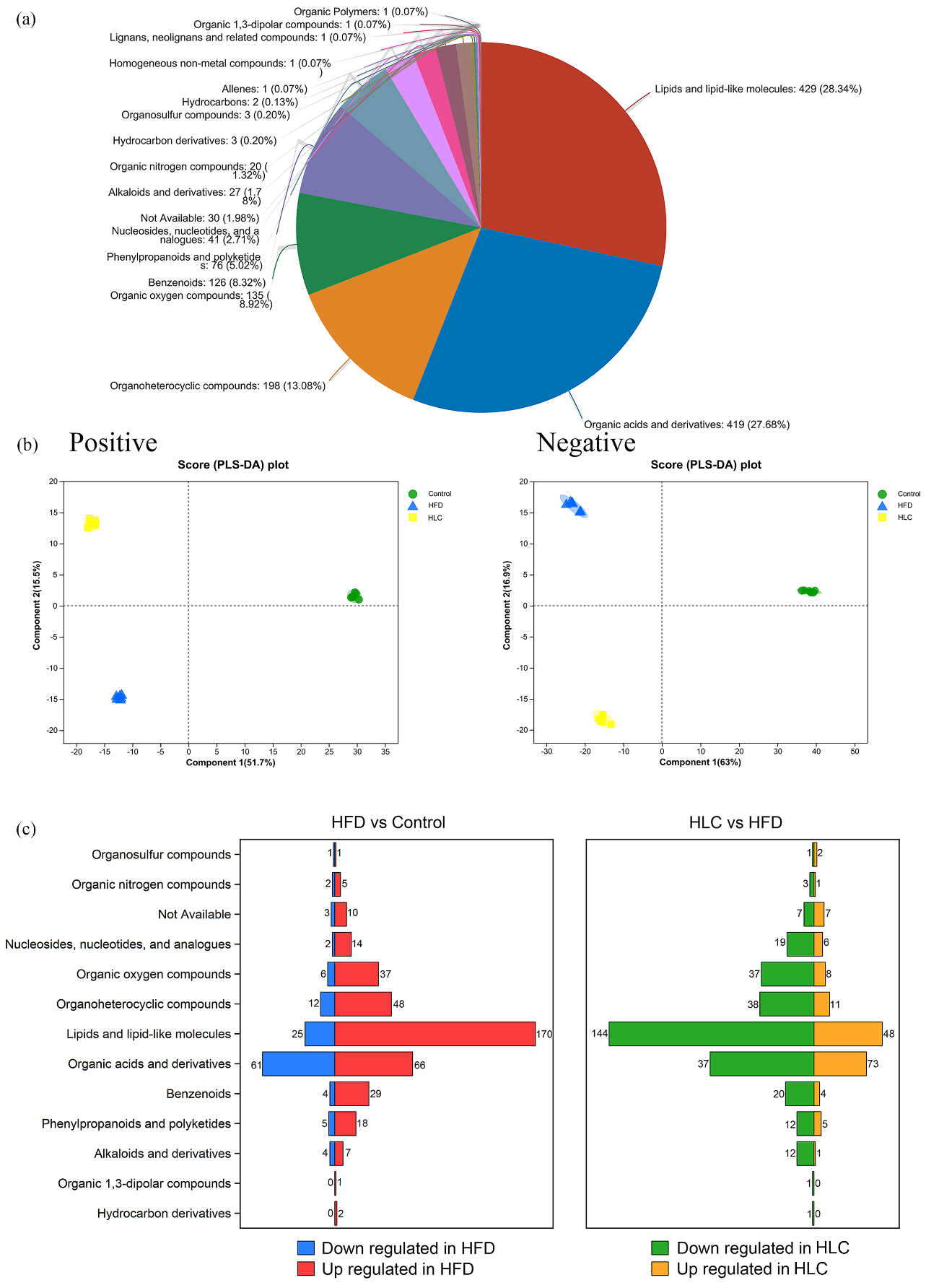


**Figure S3**. Effect of L-carnitine on the intestinal metabolite composition of high-fat-fed carp. (a) The pie chart of classified metabolite composition; (b) The plot of PLS-DA scores in cationic and anionic mode; (c) Differences between groups for different types of metabolites.
